# PD66 Real-World Evidence In Healthcare Macro-Management: Judicial Access To Pembrolizumab-Axitinib For Advanced Kidney Cancer In Uruguay (2020 to 2022)

**DOI:** 10.1017/S0266462325101980

**Published:** 2025-12-29

**Authors:** Krizia Osterkamp, Daniel Pedrosa, Abayubá Perna, Eliana Lanzani

## Abstract

**Introduction:**

In recent years, there has been an increase in judicial claims in Uruguay seeking pembrolizumab-axitinib for the treatment of advanced kidney cancer. Understanding the characteristics and clinical evolution of patients accessing this medication through judicial rulings is essential to support health policies based on real-world evidence (RWE).

**Methods:**

This study aimed to describe the sociodemographic and clinical characteristics, as well as global survival, of patients diagnosed with advanced clear cell renal carcinoma who received pembrolizumab-axitinib through judicial claims against the National Resources Fund (Fondo Nacional de Recursos) in Uruguay between 2020 and 2022. Patients with a favorable judicial ruling during the study period who received at least one dose of the combination therapy were included. Descriptive analyses of sociodemographic and clinical characteristics were conducted, and survival was estimated using the Kaplan-Meier method.

**Results:**

Between 2020 and 2022, 46 patients received pembrolizumab-axitinib treatment through judicial claims for advanced clear cell renal carcinoma. The average age of the patients, who were predominantly male, was 55.6 years. Most patients were from the private healthcare sector, with a similar distribution between the capital and the interior of the country. The survival analysis showed a global survival rate of 84.8 percent at 12 months (95% confidence interval [CI]: 70.7, 92.4), 78.3 percent at 24 months (95% CI: 61.8, 88.3), and 67.1 percent at 36 months (95% CI: 38.8, 84.5).

**Conclusions:**

This real-world study provided promising results regarding the effectiveness of pembrolizumab-axitinib treatment in the Uruguayan healthcare context. The findings favored the opportunity to incorporate this therapy for advanced kidney cancer into the Comprehensive Health Care Plan. Additionally, it highlighted the importance of RWE in supporting healthcare policy decisions.

